# Multiscale and multimodal imaging for three-dimensional vascular and histomorphological organ structure analysis of the pancreas

**DOI:** 10.1038/s41598-024-60254-9

**Published:** 2024-05-02

**Authors:** Gabriel Alexander Salg, Verena Steinle, Jonas Labode, Willi Wagner, Alexander Studier-Fischer, Johanna Reiser, Elyes Farjallah, Michelle Guettlein, Jonas Albers, Tim Hilgenfeld, Nathalia A. Giese, Wolfram Stiller, Felix Nickel, Martin Loos, Christoph W. Michalski, Hans-Ulrich Kauczor, Thilo Hackert, Christian Dullin, Philipp Mayer, Hannes Goetz Kenngott

**Affiliations:** 1 Clinic for General-, Visceral- and Transplantation Surgery, University Hospital Heidelberg, Im Neuenheimer Feld 420, 69120 Heidelberg, Germany; 2 Clinic for Diagnostic and Interventional Radiology, University Hospital Heidelberg, Im Neuenheimer Feld 420, 69120 Heidelberg, Germany; 3 Division of Radiology, German Cancer Research Center (DKFZ), Im Neuenheimer Feld 280, 69120 Heidelberg, Germany; 4 Institute of Functional and Applied Anatomy, Hannover Medical School, Carl-Neuberg-Str. 1, 30625 Hannover, Germany; 5 Translational Lung Research Center, Member of the German Center for Lung Research, University of Heidelberg, Im Neuenheimer Feld 130.3, 69120 Heidelberg, Germany; 6 Hamburg Unit, European Molecular Biology Laboratory, c/o Deutsches Elektronen-Synchrotron DESY Hamburg, Notkestr. 85, 22607 Hamburg, Germany; 7 Department of Neuroradiology, University Hospital Heidelberg, Im Neuenheimer Feld 400, 69120 Heidelberg, Germany; 8 Clinic for General-, Visceral- and Thoracic Surgery, University Medical Center Hamburg-Eppendorf, Martinistr. 52, 20246 Hamburg, Germany; 9 Institute for Diagnostic and Interventional Radiology, University Medical Center Goettingen, Robert-Koch-Str. 40, Goettingen, Germany; 10 Translational Molecular Imaging, Max Planck Institute for Multidisciplinary Sciences, Hermann-Rein-Str. 3, Göttingen, Germany; 11 Present Address: Medical Faculty, Heidelberg University, Heidelberg, Germany

**Keywords:** Pancreas, Imaging, Synchrotron, Vascularization, Virtual histology, Computed tomography, Islets of Langerhans, Imaging, X-ray tomography, Pancreas

## Abstract

Exocrine and endocrine pancreas are interconnected anatomically and functionally, with vasculature facilitating bidirectional communication. Our understanding of this network remains limited, largely due to two-dimensional histology and missing combination with three-dimensional imaging. In this study, a multiscale 3D-imaging process was used to analyze a porcine pancreas. Clinical computed tomography, digital volume tomography, micro-computed tomography and Synchrotron-based propagation-based imaging were applied consecutively. Fields of view correlated inversely with attainable resolution from a whole organism level down to capillary structures with a voxel edge length of 2.0 µm. Segmented vascular networks from 3D-imaging data were correlated with tissue sections stained by immunohistochemistry and revealed highly vascularized regions to be intra-islet capillaries of islets of Langerhans. Generated 3D-datasets allowed for three-dimensional qualitative and quantitative organ and vessel structure analysis. Beyond this study, the method shows potential for application across a wide range of patho-morphology analyses and might possibly provide microstructural blueprints for biotissue engineering.

## Introduction

The pancreas is characterized primarily by its dual function both as exocrine and endocrine gland of complex histo-morphological structure. While exocrine acini secrete aggressive digestive enzymes into the pancreatic ductal system, fragile endocrine cell clusters, namely the islets of Langerhans, are spatially dispersed throughout the organ. At first glance this phenomenon, which is fundamentally similar across vertebrate species, might seem paradox^1,2^. However, research has shown that complex interactive communication systems referred to as exocrine-endocrine-ductal axis influence pancreatic function^3–5^. Studies on exocrine and endocrine pancreatic diseases buttressed communication between these traditionally separately investigated organ regions^4–8^. Further studies demonstrated that in addition to autocrine and paracrine signaling, the vascular system acts as mediator for systemic communication within the organ^3,4,9^. Recent findings of a functional, bi-directional blood flow between endocrine and exocrine sections, emphasize possible further interdependencies and interactions of the organ sections in pancreatic pathologies^3,4,6^. Vascular transport of insulin and other signals such as cholecystokinin from endocrine cells to exocrine tissue or its malignant degenerations and reciprocal delivery of possibly tumor-derived factors, might be worth exploring considering their disruptive consequences^6,10,11^. Further, the pancreatic endocrine function is based on a close interaction between Langerhans islets and their vascular system^3,12,13^. These blood vessels are essential for physiological islet function. Eventhough the endocrine pancreas represents only 1–2% of the organ volume, it accounts for 5–15% of pancreatic blood flow^2^. Besides being prerequisite for blood glucose transport to islets and e.g., insulin transport away from islets into the system, a complex, yet not fully understood, system of interactions was described to alter the function of endocrine cells. Studies have described structural alterations of the islet microvasculature in pathologies such as type 1 or type 2 diabetes^2,14,15^. However, etiological sequences and functional consequences are to be specified.

Therefore, a more versatile understanding of the whole-organ vascular network is necessary. 3D imaging enables anatomical features to be studied in a full, spatial context^16,17^. At a macroscopic level, clinical computed tomography (CT) and digital volume tomography (DVT) allow for 3D, volumetric tissue imaging and are applied in clinical routine. At the microscopic tissue and cellular level, imaging is predominantly constrained to 2D-examinations^18^. Standard histology tissue sections provide 2D snapshots of specific features and require destruction of the sample in the imaging process. Micro computed tomography (µCT) is conceptually equivalent to clinical computed tomography^18^. The sample is placed in a X-ray beam and absorption patterns from a high number of rotation angles are detected to allow for volumetric reconstruction^18^. However, the intrinsically low X-ray absorption contrast in soft tissues often requires preparation steps that increase the radio-opacity of the sample^18–21^. The mesoscopic resolution that can be achieved by µCT imaging allows to assess 3D spatial relationships that non-serial histological sections for microscopy cannot provide. At the same time, the relatively large field of view of common µCT systems enables correlation of imaging findings with clinical volumetric imaging^20^. Finally, synchrotron-based propagation-based imaging (PBI) allows the high-resolution 3D-examination of soft tissues at the (sub-)cellular microscopic level^21^. Thus, non-destructive virtual histology of tissue samples is possible without interference with subsequent examinations or histological processing^21^.

In this study, we present a methodological process that might serve as basis for a 3D morpho-functional digital twin of the pancreas with special emphasis on the pancreatic vascular network. This imaging process provides quantitative three-dimensional information from a macroscopic to a microscopic level thereby bridging the gap between clinical routine examinations and high-resolution ex vivo imaging in a research setting. The resulting multiscale, multimodal datasets can be applied in further in silico experiments e.g., using fluid dynamics for blood flow and biochemical process simulations to improve the understanding of interactions on a whole-organ level in health and disease.

## Results

### Development of a multiscale and multimodal imaging method

**Figure 1:**
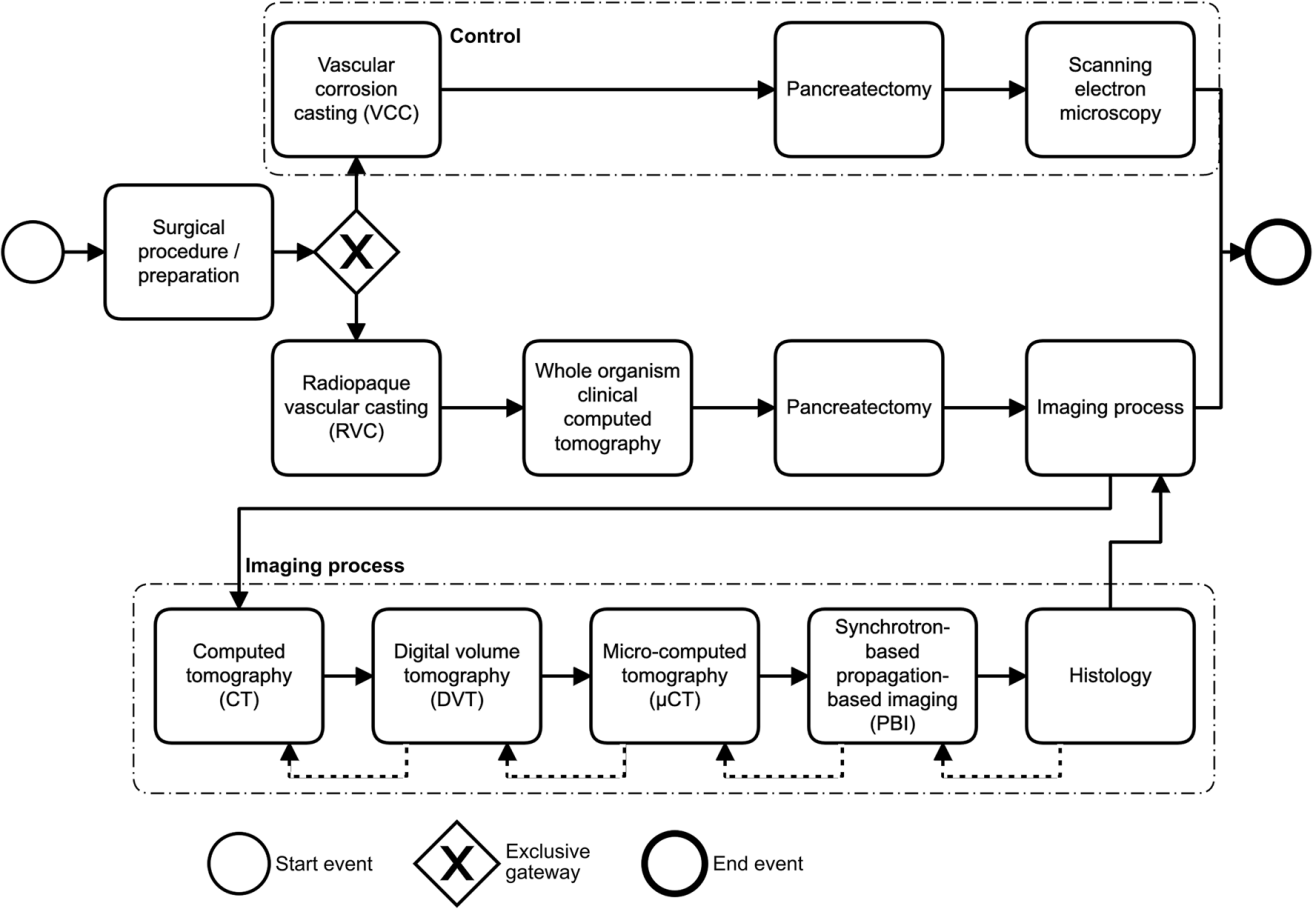
Experimental data acquisition workflow (Business Process Model and Notation (BPMN) 2.0). After surgical preparation either vascular corrosion casting (VCC) or radiopaque vascular casting (RVC) were performed. VCC with subsequent tissue corrosion and data acquisition by scanning electron microscopy served as a control in this study. In RVC, the whole animal was imaged by clinical computed tomography before resection of the pancreas. Pancreatic tissue and enduring intravascular contrast were further investigated using an imaging process consisting of computed tomography (CT), digital volume tomography (DVT), micro-computed tomography (µCT), synchrotron-based propagation-based imaging (PBI) and correlating histology. Within the imaging process, data correlation can be achieved with the previous, lower resolution modality (dashed arrows).

**Figure 2:**
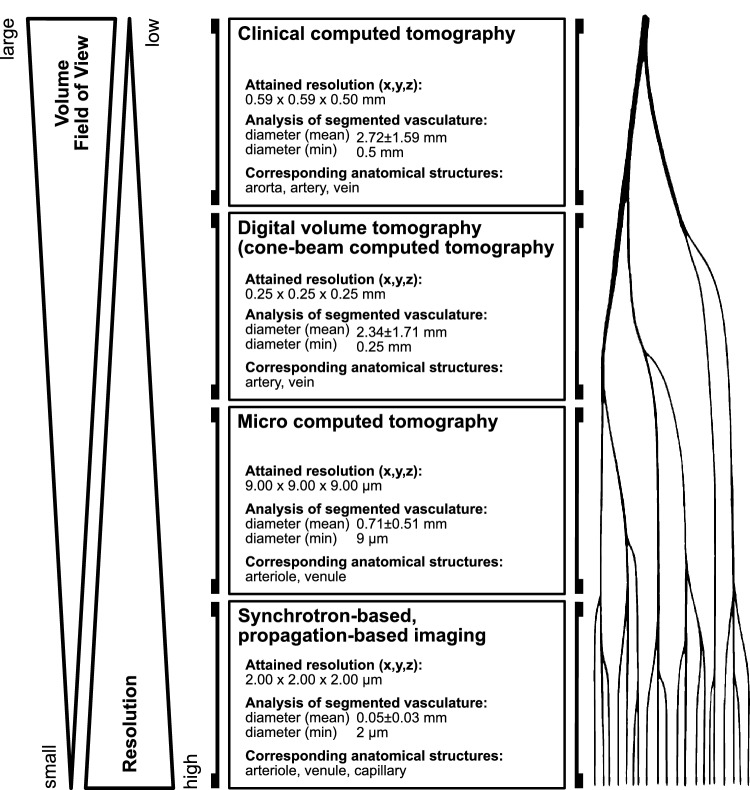
Multiscale, multimodal imaging process method. Clinical CT and DVT of the whole pancreas detected vascular structures with a mean diameter of 2.72 mm and 2.34 mm, respectively. µCT imaging with a smaller FOV and a resolution of 9.00 µm iso-voxel edge length allowed to detect arterioles and venules with a mean vessel diameter of 0.71 mm in the respective volume. Synchrotron-based PBI partially captured capillaries and resulted in a mean vessel diameter of 0.05 mm in the investigated volume.

In this study, a multiscale and multimodal imaging process was established. The imaging process is depicted in Fig. 1. Proof of concept and feasibility of the method was demonstrated using porcine pancreatic specimen. Radiopaque vascular casting (RVC) of the vasculature of the specimen was conducted. A control was performed by conventional vascular corrosion casting (VCC; intravascular casting and subsequent corrosion of tissue) with investigation of the remaining vascular structures by scanning electron microscopy. The imaging process allows the investigation of pancreatic soft tissue specimen of different volume dimensions (field of view, FOV) from the full body level to small tissue fractions in the µm^3^ scale (Fig. 2). The FOV of imaging modalities correlated inversely with the attainable resolution (voxel edge length, Fig. 2). RVC permanently stained vascular structures for x-ray-based imaging, while at the same time avoiding any influence on cellular integrity necessary for immunohistochemical staining (macroscopic depiction of in situ casting of porcine pancreas s. Supplementary Figure S1). Image-guided tissue sections, that were immunolabeled, were correlated with PBI 3D-data. Segmented 3D vascular networks were converted to surface meshes and volume meshes used for branching analysis. Comprehensive vessel diameter analyses of the 3D-datasets were performed. Finally, evaluation algorithms for clustering of retrieved vascular networks were applied for future translational studies using the methodology presented here. This study was performed in a descriptive, qualitative manner.

### Clinical computed tomography and digital volume tomography demonstrate successful vascular casting and maximum field of view

**Figure 3:**
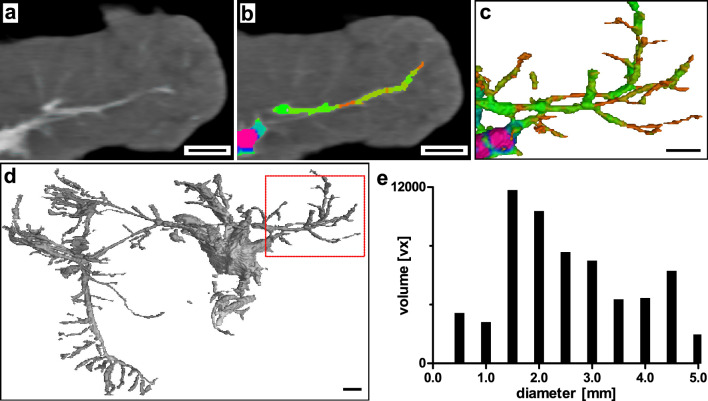
Clinical computed tomography. (**a**) Splenic lobe of porcine pancreas with RVC. Axial plane, filtered back projection. (**b**) Overlay with color-coded vessel diameter analysis. (**c**) Section of 3D-reconstruction of segmented vascular network (color-coded vessel diameter analysis). (**d**) 3D-reconstruction of whole segmented pancreas vascular network (dashed section: (**c**)). (**e**) Volume distribution of segmented vascular network by vessel diameter. Scale bar 10 mm (**a**–**d**).

CT imaging provided an overall spatial context of the whole pancreas at a relatively low resolution (full body level: Supplementary Video S1, organ level: Fig. 3). RVC enabled detection of major peri- and intra-pancreatic vessels. Small branches as secondary inter-lobular vessels were partially detectable. Analysis of vessel diameter and respective distribution of segmented vascular volume shows a maximum of vessels in the diameter range of 1.5 mm (Fig. 3e). However, based on full organ FOV and relatively low resolution, mostly large peri- and intra-pancreatic vessels were detected with a mean vessel diameter of 2.72 mm (± 1.59, standard deviation (SD)) (Fig. 2). A 3D-surface mesh of a vascular network detected by CT imaging can be found in the supplemental to this article (Supplementary File S1). It is important to note that the voxel edge length achievable with this technique was 0.59 × 0.59 × 0.50 mm. Structures that could be identified with clinical computed tomography correspond to larger arteries and veins.

**Figure 4:**
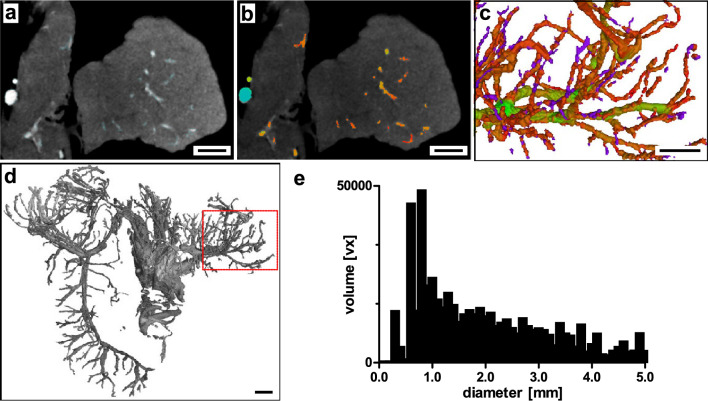
Digital volume tomography. (**a**) Splenic lobe of porcine pancreas with RVC. Axial plane. (**b**) Overlay with color-coded vessel diameter analysis. (**c**) Section of 3D-reconstruction of segmented vascular network (color-coded vessel diameter analysis). (**d**) 3D-reconstruction of whole segmented pancreas vascular network (dashed section: (**c**)). (**e**) Volume distribution of segmented vascular network by vessel diameter. Scale bar 10 mm (**a**–**d**).

DVT imaging generated a dataset with 0.25 mm iso-voxel edge length covering the whole organ volume (Fig. 4). Eventhough comparable to computed tomography by covering larger peri- and intra-pancreatic arteries and veins with a mean vessel diameter of 2.34 mm (± 1.71, SD), DVT enabled detailed identification of secondary vascular structures branching from main intra-pancreatic vessels (Figs. 2, 4a–c). In addition, detailed inspection and segmentation of contrast-enhanced vascular structures e.g., in the splenic lobe of the porcine pancreas identified concomitant vascular structures, presumably artery and vein (Supplementary Figure S2). Vessel diameter analysis revealed vascular structures with a minimum diameter of 0.25 mm and volume distribution by vessel diameter shows a shift towards smaller vessels (peak 0.8 mm) (Fig. 4e). A 3D-surface mesh of a vascular network detected by DVT imaging can be found in the supplemental material to this article (Supplementary File S2). Based on DVT imaging, volumes of interest (VOI) were defined for µCT imaging to enable tracing of structures across length scales of hierarchical imaging.

### Micro-computed tomography (µCT) is a mesoscopic link in the process hierarchy and detects pre-capillary vascular structures

**Figure 5:**
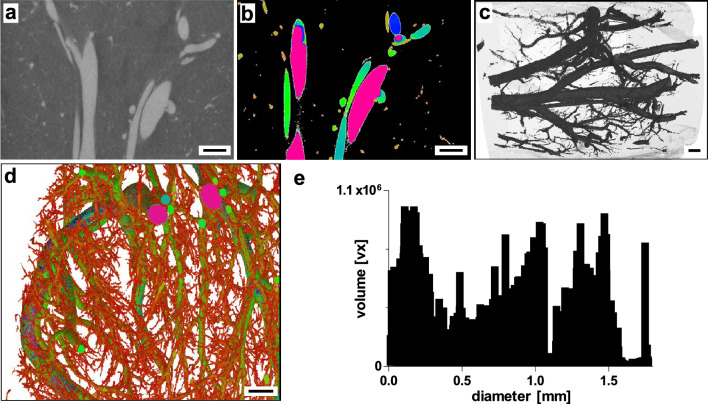
Micro-computed tomography. (**a**) Tissue section of splenic lobe of porcine pancreas with RVC. Axial plane. (**b**) Segmented vascular network (color-coded vessel diameter analysis, axial plane). (**c**) Section of 3D-reconstructed dataset (translucent). (**d**) Section of 3D-reconstruction of segmented vascular network (color-coded vessel diameter analysis). (**e**) Volume distribution of segmented vascular network by vessel diameter. Scale bar 2 mm (**a**–**d**).

µCT imaging generated a dataset of 9.00 µm iso-voxel edge length (here: splenic lobe, Fig. 5). Uniform intravascular filling by the casting agent was found in small-scale vasculature branching from large scale arterial and venous structures (Fig. 5a–c). Minor discontinuances of RVC were remodeled using a hole-filling algorithm. Concomitant progression of vascular structures as already detected in DVT imaging for large-scale arteries and veins, were pursuable to a small-scale inter-septal level (Fig. 5a–d, Supplementary Figure S2). Vessel diameter analysis of the investigated VOI resulted in a bimodal distribution with one volume maximum at the high end (peak at 1.47 mm) and one maximum at the lower end with an intraluminal diameter of 0.11 mm (Fig. 5e). A mean vessel diameter was calculated with 0.71 mm (± 0.51, SD) in the investigated section (Fig. 2). A 3D-surface mesh of a vascular network detected by µCT imaging can be found in the supplemental to this article (Supplementary File S3). These results implicate that µCT imaging can detect small-scale arteries and veins as well as arterioles and venules with a minimum vessel diameter of 9.0 µm. µCT imaging did not achieve resolutions to identify capillary structures and morphological analysis of the segmented vascular network did not reveal distinguishable networks that might correspond to endocrine tissue vasculature. However, FOV and spatial resolution of µCT imaging allows to bridge a gap between clinical volumetric imaging and high-resolution microscopic volumetric imaging by means of traceability.

### Synchrotron-based propagation-based imaging (PBI) identifies capillary structures

**Figure 6:**
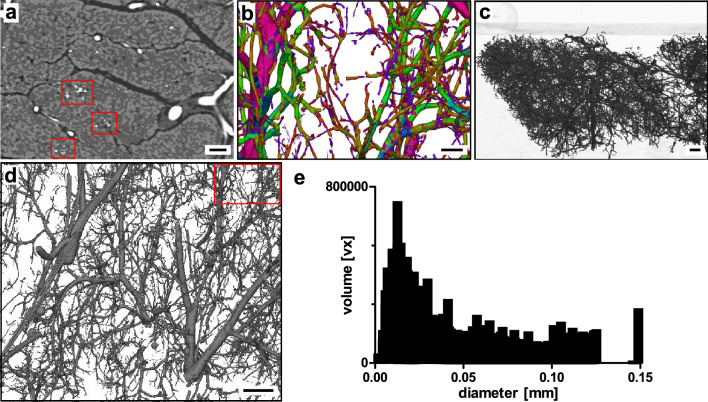
Synchrotron-based propagation-based imaging (PBI). (**a**) Tissue section of splenic lobe of porcine pancreas with RVC; axial plane. Highly vascularized areas can be identified in defined soft-tissue sections (dashed sections). (**b**) Section of 3D-reconstruction of segmented vascular network (color-coded vessel diameter analysis). (**c**) 3D-reconstruction of segmented vascular network in translucent paraffin (upper left corner: surface marker). (**d**) Section of 3D-reconstruction of segmented vascular tree (dashed section: (**b**)). (**e**) Volume distribution of segmented vascular network by vessel diameter. Scale bar 100 µm (**a**,**b**), 500 µm (**c**,**d**).

Synchrotron-based PBI of tissue sections of the splenic lobe of the porcine pancreas was conducted after tissue dehydration and paraffin embedding. Paraffin-embedding was chosen due to its compatibility with further histological processing. FFPE specimens in histology cassettes were directly used for non-invasive imaging. It is worth noting, that uniform shrinkage of the intravascular casting agent was observed, which can be attributed to dehydration during paraffin-embedding process^17,22^. Synchrotron-based PBI had the smallest FOV, however, achieving the highest resolution with an iso-voxel edge length of 2.0 µm. Application of this imaging technology allowed identification of inter- and intralobular arterioles and venules together with capillary structures and, thus, the dense vascular network in pancreatic tissue (Fig. 6). Concomitant arterial and venous structures could be traced to arterioles and venules running together up to a small-scale inter-septal level (Supplementary Figure S2). Next-order branching arterioles and venules penetrating and supplying exocrine lobules did not run parallel anymore but independently. Certain, distinct areas of pancreatic soft tissue showed a high density of small vascular structures, supposedly corresponding to endocrine portions (Fig. 6a, dashed sections). Analysis of volume distribution by vessel diameter showed a distinct peak at 12.0 µm in the respective VOI (Fig. 6e). The mean vessel diameter by vessel volume was 0.05 mm (± 0.03, SD) with a minimally detectable diameter of 2.0 µm (Fig. 2). A 3D-surface mesh of a vascular network detected by PBI can be found in the supplemental to this article (Supplementary File S4).

### Tissue sections can be correlated to PBI datasets for virtual histology

**Figure 7:**
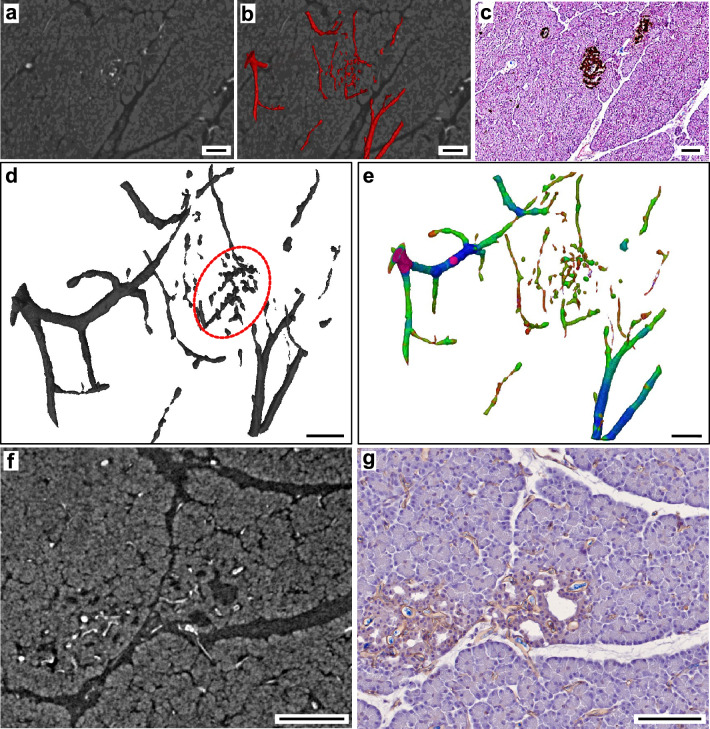
Synchrotron-based propagation-based imaging (PBI) correlated with tissue sections stained by immunohistochemistry. (**a**) Tissue section with intravascular radiopaque casting, axial plane. (**b**) Tissue section with segmented intravascular radiopaque casting (red), axial plane. (**c**) Tissue section with anti-insulin staining (brown), intravascular casting agent remnant (blue) and hematoxylin counterstaining. (**d**) 3D-reconstruction of segmented peri- and intra-islet vascular structures (dashed section: intra-islet). (**e**) Vessel diameter analysis of 3D-reconstruction of segmented peri- and intra-islet vascular structures. (**f**) Tissue section with intravascular radiopaque casting, axial plane. (**g**) Tissue section with anti-CD31 staining to label endothelium (brown), intravascular casting agent remnant (blue) and hematoxylin counterstaining. Scale bar 100 µm (**a**,**b**,**c**,**f**,**g**), 200 µm (**d**,**e**).

**Figure 8:**
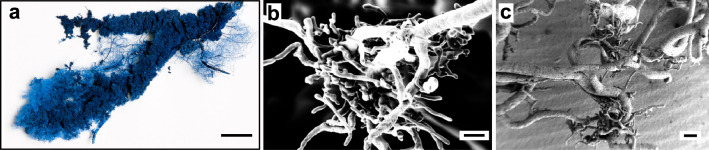
Scanning electron microscopy (SEM) of VCC. (**a**) Macroscopic image. Blue Biodur® casting agent, intravascular application. (**b**) SEM section of corrosion cast depicts a vascular bundle, presumably intra- and peri-islet vasculature. (**c**) SEM section of corrosion cast depicting vascular bundles, presumably intra- and peri-islet vasculature. Scale bar 20 mm (**a**), 40 µm (**b**), 100 µm (**c**).

By application of RVC, low-density differences of soft tissues even far from the central axis of rotation were addressed and investigations of peri- and intra-insular vascular networks by non-destructive imaging were performed (Fig. 7). Confirmation is achieved by correlation of PBI data (Fig. 7, Supplementary Figure S3) with corresponding tissue sections stained by immunohistochemistry (Fig. 7c). Immunolabeling of vascular endothelium by CD31-staining in histological sections confirmed successful vascular casting by RVC and was correlated to PBI 3D-datasets (Fig. 7f,g, Supplementary Figure S3). In PBI data, highly vascularized volumes, disseminated throughout the specimen, were found. Corresponding tissue sections and positive immunohistochemical staining for insulin at these locations verified them as islets of Langerhans. A 3D-surface mesh of the peri- and intra-islet vascular network detected by PBI imaging can be found in the supplemental to this article (Supplementary File S5). VCC has been widely used to study vascular morphology and vascular networks^23,24^. Here, VCC of the pancreatic vasculature was used as a control (Fig. 8). Scanning electron microscopy (SEM) of the casting agent, that remained after complete dissociation of tissue, showed distinct lesions of high vascular density (Fig. 8b,c) spatially dispersed in the entire cast. As described in the literature, such areas might correspond to endocrine cell clusters, namely islets of Langerhans^23,24^ .Vessel diameters of presumed intra-islet vascular structures were similar to vessel diameters of intra-islet vasculature detected by PBI. However, proof by means of specific immunostaining as demonstrated above cannot be achieved due to the nature of the VCC method. Additionally, with 3D-SEM being technically demanding and time-consuming, this method is practically limited to 2D-imaging of small sectors of the specimen.

### Generation and arborization analysis for characterization of vascular network

In future translational approaches, data obtained by using this imaging process might be useful to identify and analyze vascular alterations in selected pathologies. The application of evaluation algorithms for complex vascular networks is necessary for objective comparison of different samples and entities. The methodology to perform such evaluations was adapted to our exemplary datasets here and is elaborated below. Previously developed analysis tools and software workflows for lung vasculature by Grothausmann et al. and Labode et al. were adapted for application of the pancreatic vascular network datasets (RVC)^25,26^. Digital segmentation of representative, uninterrupted vessels attained from µCT imaging and PBI were reduced to 2D-graphs (Supplementary Figure S4). Vessel segments were defined as either segments between two bifurcations or segments between a bifurcation and a terminal node. In total 954 distinct vessel segments were identified in a single, continuous vessel obtained from µCT imaging and 1025 distinct segments in a single, continuous vessel obtained from the PBI dataset. The analysis of a larger vascular network from a FFPE tissue section that was obtained by PBI resulted in 35,524 distinct vessel segments. Resolution is the limiting factor. The higher resolution of the PBI dataset allows identification of small-scale vasculature and further bifurcations beyond the obtainable resolution in the µCT dataset. Generation, order and Strahler order algorithms as vascular branching classifications were applied to the respective 2D datasets^27–29^. In a first generational approach the graph root node is attributed generation 1^27^. Each daughter branch after every bifurcation increases the generation by 1^27^. The order approach starts attributing identifiers at the terminal nodes (last bifurcation of smallest vessel segments)^28^. Backwards analysis towards the root node increments the highest order by one every time two or more vessels meet^28^. The Strahler order modifies the conventional order algorithm by only increasing the order number towards the root node by one if two or more branches of the same order merge^29^. Otherwise, the highest order continues^29^. The Strahler order approach resulted in 6 groups in which the single vessel from µCT and PBI can be categorized. Analysis of the vascular network of a larger FFPE tissue section revealed a vascular network categorized in 8 Strahler orders. Detailed results of the specimen analysis using the above-described evaluation algorithms can be found in Supplementary Table S1 in the supplement to this article. By plotting the group attribution following the above-described algorithms against the parameters of vessel segment diameter and vessel segment length, the variance within each group was visualized (Supplementary Figure S5).

**Figure 9:**
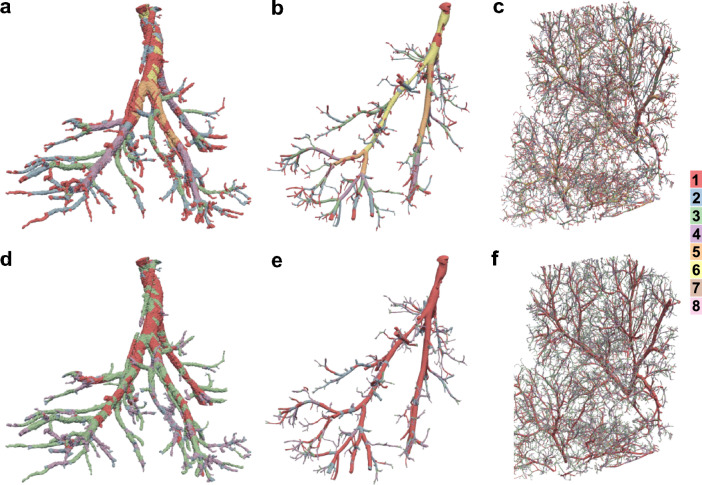
3D-reconstruction of digitally segmented vascular structures with color-mapping of vessel segment categorization according to (**a**–**c**, top row) Strahler order and (**d**–**f**, bottom row) GMM clustering (right: color-legend for vessel segment categorization). (**a**,**d**) Single, continuous vessel obtained from µCT imaging. (**b**,**e**) Single, continuous vessel obtained from PBI. (**c**,**f**) Vascular network obtained from PBI. (**a**,**b**) 6 Strahler orders, (**c**) 8 Strahler orders, (**d**,**e**) 4 GMM clusters, (**f**) 5 GMM clusters.

An alternative method to group segments of such vascular networks was achieved by clustering using a Gaussian mixture model (GMM)^30^. To determine the optimal number of clusters for each dataset the Bayesian information criterion (BIC) was calculated for the cluster counts 1 to 9 and 14 geometric constellations. Both single vessels (µCT and PBI) had a maximum BIC value at 4 clusters (Supplementary Figures S6a,b, S4j,k). Consequentially, the number of clusters for GMM was set 4. Attribution of each data point to their respective cluster was calculated using the GMM. For the vascular network of a whole FFPE tissue section obtained from PBI, a BIC of 5 was calculated and data points were attributed to 5 clusters using a GMM (Supplementary Figure S6c, S4l). The Davies-Bouldin index and Dunn index were calculated to evaluate the performance of the clustering compared to conventional grouping algorithms (Supplementary Figure S6d-i, Supplementary Table S2). In all investigated specimens, GMM clustering resulted in lowest Davies-Bouldin index and highest Dunn index indicating well-separated clusters, in most cases followed by Strahler order. Detailed analysis of the vessel segment categorization including mean, minimum and maximum vessel segment diameters and total vessel segment volumes for Strahler order and GMM clustering can be found in Supplementary Table S3 in the supplement to this article. Figure 9 depicts mapping of categorization results from Strahler order and GMM clustering back on the 3D-vessels. Qualitative evaluation shows that the Strahler order correlated well with the respective vessel segment diameters resulting in coherent vessel groups (Fig. 9a–c). The GMM clustering is based on vessel segment diameter and vessel segment length. Despite superior results were achieved in Davies-Bouldin and Dunn index, mapping back the GMM cluster attribution to the 3D renderings (Fig. 9d–f) showed less sharp categorization of arborization especially in the µCT dataset (Fig. 9d). As described before, application of different classification algorithms can lead to dissimilar results and different levels of internal morphometric similarity^26^. This shortcoming might be overcome by addition of other input parameters. Labode et al. used wall thickness of the respective segments as an additional parameter^26^. Resolution and contrast of the soft tissue specimen without RVC in this study did not allow investigation of this parameter in µCT or PBI data.

## Discussion

The aim of this study was to establish a process that enables multiscale and multimodal investigation of biological specimen from a macroscopic whole organism level in a porcine animal model to a microscopic level of cellular resolutions detecting islets of Langerhans as well as peri- and intra-islet vascular structures (Supplementary Video S1). With PBI as a last step in the imaging process, high-resolution data were obtained that could be correlated with histology. The particular advantage of PBI is the generation of a 3D-dataset in non-destructive fashion that extends classical two-dimensional histology approaches. Frohn et al. previously investigated pancreatic specimen by means of µCT and PBI and demonstrated the capability of PBI to identify and segment islets of Langerhans in an unstained FFPE sample^31^. Although multiscale imaging was achieved using two entities, no clinical imaging technologies were included. In contrast to our study, vascular networks were not investigated. Especially 3D-datasets of vascular networks specific for organotypic morpho-functional units can contribute to understanding interactions between endocrine and exocrine units mediated by the vascular system and alterations of the networks in several benign and malignant pathologies. Further, PBI data from this study showed, that identification of islets of Langerhans is possible based on analysis of the vascular network. The intra-luminal contrast of dense intra-islet vascular networks could be distinguished from vascular networks of the exocrine pancreas. Eventhough capillary structures were identified, a uniform end-to-end representation of the capillary bed could not be achieved (e.g., Figs. 6b, 7d,e). This limitation might be caused either by incomplete filling with casting agent or by insufficient resolution. The experimental design of this study required RVC to achieve adequate contrast for differentiation of vascular structures in soft tissue. Previous studies using µCT or PBI showed that depending on the tissue type staining is not always necessary for vascular structure segmentation^32^. Investigating the vascular supply of the human spiral ganglion in bony cochlea using PBI was achieved without tissue staining based on intrinsic contrast of different tissue densities^32^. However, in this study a spatial resolution of approximately 15 µm was achieved; thus, imaging and analysis of capillary structures was not possible^32^.

This study has limitations. First, porcine pancreata were investigated for early-stage methodological experimental science. Histomorphology and function of porcine pancreata are highly similar to their human equivalents^33,34^. In contrast to human anatomy, a single pancreatic duct communicates with the duodenal lumen distal to and separated from the common bile duct^35^. The macroscopic morphology is more lobulated and fattier. Thus, future studies investigating human pancreatic specimen and respective pathologies are feasible and necessary^16^. Reprocessing of tissue for histology resulted in shrinkage and dissolution of the casting agent. Intra-vascular staining is not necessary for evaluation of histological staining. However, shrinkage of the radiopaque casting agent also occurs, to a lesser extent, during tissue formalin-fixation and paraffin-embedding for non-destructive imaging and might lead to underestimation of vessel diameters. Quantitative data of casting agent shrinkage in several casted tissue specimens will allow to extrapolate a shrinkage factor. Methodologically, artificial intelligence tools might be able to replace semiautomatic segmentation using region-growing tools^36,37^. Such fully automatic segmentation tools are subject to ongoing research and not focus of this current study. In theory, FOV and attainable resolution of the respective modalities in this imaging process allow traceability of structures from clinical volumetric imaging at a whole organism level to a microscopic, cellular level and even immunolabeled sections. However, a complete multiscale and cross-entity data integration beyond manual correlation can only be achieved by exact dynamic transformation of inter-modality data due to deformations of the sample. Apart from the mathematical difficulty, computer power necessary to perform such transformations on large three-dimensional datasets will exceed infrastructural capacities especially from a clinical or translational perspective and might require application of additional guide structures^38^. A recently developed technology named hierarchical phase-contrast tomography (HiP-CT) using the Extremely Brilliant Source of European Synchrotron Radiation Facility (ESRF) enables high-resolution imaging across length scales without physical subsampling and the necessity of data registration^39^. However, this method is currently available only at this single synchrotron facility and even though being inherently data efficient, still requires massive computing power. As alternative methodologies to PBI, optical imaging systems such as plane illumination and serial two-photon microscopy have traditionally been used for investigating 3D vascular networks^40,41^. By application of the ‘Extended-volume imaging system’, Kelch et al. visualized and analyzed the entire vascular network of a lymph node^40,42^. Combination of 63,706 confocal images at 2.0 µm pixel resolution resulted in a total reconstructed volume of 3.88 mm^3^^40^. Shortcomings of this optical approach include the time-consuming imaging (2 weeks for lymph node specimen) and the destruction of the whole sample during the imaging process^40^. Interestingly, the Kelch et al. found a mean vessel diameter of 13.47 µm in the studied lymph node^40^. Vessel diameter analysis in our PBI dataset of the pancreatic vascular network revealed a peak at a diameter of 12 µm. Tissue clearing methods such as CLARITY or T3 and subsequent confocal or two-photon microscopy enabled non-destructive 3D-data acquisition of pancreatic vascular networks and surrounding microenvironment for specimen of up to 1 mm thickness^43–45^. Such transparent tissue imaging methods demonstrate superior results in the ability to discriminate endocrine and exocrine tissue while at the same time maintaining vascular network visualization possibilities^46^. More recently, optical clearing of whole organs was achieved; however, such methods require several months, volume-suitable high-resolution three-dimensional read-outs are lacking, staining is dependent on antibody penetration depth and extremely large datasets have to be processed^44^. In contrast, the high penetration power of X-rays, e.g. in PBI, achieves a high ratio of object thickness to spatial resolution^47^.

The data generated in this study provide hierarchical information on 3D morphology and spatial relationships across length scales and might contribute to current global initiatives such as NIH’s Human Biomolecular Atlas Program envisioning a high-resolution atlas of the human body^48^. Our whole dataset may serve as a basis for a digital twin of the porcine pancreatic vascular network. Digital organ twins might facilitate in silico experiments for e.g., blood flow simulation and pharmacodynamical drug testing. In addition, if applied to e.g., exocrine or endocrine pancreatic pathologies such as pancreatic ductal adenocarcinoma, high-resolution 3D-datasets, acquired in non-invasive fashion will augment current state-of-the-art 2D data acquisition in clinical pathology and research^16^. Progress in understanding the role of microvasculature in diabetes has been achieved amongst others by application of new imaging techniques^49^. The application of evaluation algorithms and clustering methods proves the feasibility to derive metric data from the segmented imaging datasets that enable objective comparison of specimens.

In future studies, the methods developed here can be applied to study human specimen of relevant pancreatic and vascular pathologies and beyond without a confinement of tissue destruction. Lastly, state-of-the-art tissue engineering and bioartificial organ research is still limited by a lack of vascular networks in scaled constructs^50–52^ Possibilities of analyzing high-resolution vascular networks as outlined here might allow to deduct and condense information to generate organ-specific rule-based frameworks for bioartificial vascular networks and subsequently three-dimensional organ blueprints^52^.

## Experimental section/methods

### Porcine animal model

In this study deceased pigs of the sus scrofa ssp. domesticus species with a mean weight of 37.3 ± 4.5 kg were included for establishing the method (n = 10). In addition, on one animal complete end-to-end data acquisition and analysis were performed. All pigs used in the experimental laboratory were managed according to German laws for animal use and care and according to the directives of the European Community Council (2010/63/EU) and ARRIVE guidelines^53^. As described by Studier-Fischer et al., pigs were euthanized with a rapid intravenous application of 50 ml of potassium chloride solution^54,55^. Death was pronounced upon cardiac arrest and an end-expiratory CO_2_ partial pressure below 8 mmHg. Immediately after death was determined, the surgical and in situ casting procedure of this study was started.

### Surgical procedure and in situ vascular casting

After gentle preparation of the abdominal aorta, it was clamped proximal from the celiac trunc and distal from the superior mesenteric artery (SMA) (Supplementary Figure S1a). The tied aortic section was dissected, and blunt button metal cannulas (inner diameter 1 mm) were inserted into celiac trunc and SMA and tightly fixed by sutures. Next, the inferior caval vein was clamped proximal to its branching to common iliac veins and distal from the caval foramen in the diaphragm. The tied section was incised and a suction device was inserted. While flushing solution (1000 ml Sterofundin ISO® by B. Braun®/50.000 I.U. Heparin) was injected under mild pressure via the arterial cannulations, blood from the respective circulatory area was collected at the venous outlet. Flushing was continued until significant blood dilution was recognized at the venous outlet. Hereafter, fixation solution (1000 ml 0.3 M HEPES solution / 1.5% paraformaldehyde / 1.5% glutaraldehyde; pH adapted to 7.35) was injected slowly via arterial cannulation. Next either vascular corrosion casting (VCC) for tissue-free vascular network examination or radiopaque vascular casting (RVC) for non-invasive imaging and subsequent histological analysis was performed. VCC was conducted using the Biodur E20® kit (Biodur Products, Heidelberg, Germany). It was freshly mixed at a ratio of 100:45 (v/v) of Biodur E20® Plus and catalyst E20. RVC was conducted using MV-120 MICROFIL® Silicone Rubber Injection Compounds (Flow Tec, Inc., Carver, MA, USA) as described before^56^. It was freshly mixed at a ratio of 40:50:10 (v/v) of MV-Compound, MV-Diluent and MV-Curing Agent. The respective casting mixtures were injected via arterial cannulations under constant manual pressure. The infusion was stopped after return of the casting agent was observed in the venous outlet and uniform discoloration of the pancreatic tissue surface was observed (Supplementary Figure S1b–d). The material initially hardened for 20 min in situ without further manipulation. In RVC, surgical procedure was stopped for computed tomography (CT) imaging of the complete animal before organ retrieval. After in situ pancreatic imaging in RVC and after curing in VCC, major and minor duodenal papilla were identified and probed by intraluminal inspection of the descending duodenum. All parts of the pancreas were mobilized including periampullary duodenum and pylorus. The portal vein was ligated and dissected at the pancreatic anulus, and the intact organ was retrieved for further processing.

### Processing of vascular corrosion casts

In VCC, the porcine pancreas was incubated for 12 h in a 40 °C water bath for further hardening. Remaining tissue was dissolved with 15% (w/v) potassium hydroxide (RT; 2 days) and the resulting corrosion cast was subsequently rinsed in water. For scanning electron microscopy (SEM) of representative samples, specimen were 10 nm gold/platinum (80:20) sputtered (Leica EM ACE 600, Leica Microsystems GmbH, Wetzlar, Germany) and analyzed by scanning electron microscopy (Zeiss Leo Gemini 1530, Carl Zeiss AG, Oberkochen, Germany). SEM images were taken at different magnifications with an accelerating voltage of 2.0 kV.

### Processing of tissue after radiopaque vascular casting

In case of RVC, the porcine pancreas was transferred to 1000 ml of 5% formaldehyde solution (Otto Fischar GmbH & Co. KG, Saarbruecken, Germany) and next steps of the imaging process were started immediately. Fixation solution was changed every third day.

### Clinical computed tomography (CT) (whole animal, whole organ)

As described above, surgical procedure was suspended for in situ imaging of the RVC-prepared pancreas. Whole organ clinical CT was performed after pancreatectomy. Different abdominal protocols were established as regarded diagnostic by two experienced radiologists on the basis of clarity and sharpness of anatomic structures and reduced artifacts of the used intravascular radiopaque casting. The clinical CT scan (SOMATOM Edge Plus, Siemens Healthineers, Munich, Germany) was performed with the following settings: 50 mAs, 70 kV, slice thickness 0.5 mm. The corresponding software from Siemens, which is also used in everyday clinical practice, was used to reconstruct the image data. Both filtered-back-projection and iterative reconstructions were created.

### Digital volume tomography (DVT) (whole organ)

In a next step of the imaging process, digital volume tomography was performed (DVT; 3DAccuitomo 170, Morita, Osaka, Japan). The scan was obtained using following settings: gantry inclination 0°, 250 µm iso-voxel edge length, tube voltage 60.0 kV, tube current 8.0 mA, exposure time 30.8 s, G_001 recon filter. Theoretically, the DVT imaging system used in this study can achieve a resolution of 80 µm iso-voxel edge length, however, with FOV smaller than a whole organ volume. Therefore this setting was not applied.

### Micro-computed tomography (µCT) (tissue sections, lobes)

As a third step of the imaging process, µCT was performed (SkyScan1176, Bruker Corp., Billerica, MA, USA). Due to the tubular table dimensions and smaller field of view, the whole organ was scanned after dissection into duodenal, splenic and connecting lobe. A first scan of each lobe was obtained with 35 µm iso-voxel edge length. Based on this first scan, a representative area in the splenic lobe was identified and further investigated using following settings: 9 µm iso-voxel edge length (8.65 µm image pixel size, 12.33 µm camera pixel size), tube voltage 65 kV, tube current 385 µA, deg 180° scan with 0.25 deg rotation steps, AL 1 mm filter, scan time 38 min. Image reconstruction was performed using the NRecon (v.1.7.4.6, Bruker microCT, 2012) software and iterative algorithms.

### Synchrotron-based propagation-based imaging (PBI) (tissue sections)

Previous to PBI, fixated and 70% ethanol-preserved tissue specimen were paraffin-embedded according to standard procedure. Therefore, pancreatic specimen were dissected into approximately 10 × 10 × 5 mm traceable numbered blocks. PBI tomography by synchrotron radiation for high-resolution image acquisition of paraffin-embedded tissue was performed using the white beam setup of the SYRMEP (SYnchrotron Radiation for MEdical Physics) beamline of the Italian synchrotron ELETTRA (Trieste, Italy) as described previously^57^. Specimen were scanned with a sample-to-detector distance of 90 mm. A 16-bit, water-cooled sCMOS camera (Hamamatsu C11440-22C ORCA-Flash 4.0 v2) was used to acquire 3600 angular distributed projections with an exposure time of 50 ms and an isotropic voxel size of 2 µm. Scans were performed in a 360° offset regime, resulting in a scanning time of 180 s (per acquisition). A 0.5 mm Si filter was used, resulting in a mean beam energy of 19.6 keV. All datasets were reconstructed using single distance phase retrieval algorithm developed by Paganin et al.^58^ with a delta over beta ratio of 100 followed by applying filtered back projection both implemented in the SYRMEP Tomo Project software (STP)^59^. To image the entire specimens, 2–3 consecutive acquisitions were performed with a stepping of 3.3 mm in vertical direction resulting in an overlap of 0.2 mm. The reconstructions were stitched together using a custom-made python script.

### Histological analysis

Subsequent to PBI tomography, paraffin-embedded tissue was sectioned with image guidance at 5 µm thickness. Endocrine cell clusters were identified by anti-insulin staining as described previously^50^. In short, a primary anti-insulin antibody (monoclonal mouse IgG, 2D11-H5, Lot# SC-8033, SantaCruz, Dallas, TX, USA), overnight 1:100 in background reducing antibody diluent (S3022, Dako, Agilent Tech., Santa Clara, CA, USA), and a polyclonal goat anti-mouse secondary antibody (Dako, Agilent Tech.) and 3-3′diaminobenzidine staining with subsequent hematoxylin counter-staining, were used. Further, vascular endothelium was labeled using an anti-CD31 antibody (overnight 1:300 in background reducing agent, monoclonal rabbit IgG; Lot# EPR17259, ab182981, Abcam, Cambridge, UK) and a polyclonal goat anti-rabbit secondary antibody (Dako, Agilent Tech.) and 3–3′diaminobenzidine staining. Whole slides were scanned at 40×*g* using a NanoZoomer S60 Digital Slide Scanner (Hamamatsu Photonics, Hamamatsu City, Japan).

### Data analysis

3D-rendering was performed using VG Studio MAX (Volume Graphics, Heidelberg, Germany, v3.4.3). Pre-processed µCT and PBI data were filtered to suppress noise using a median filter algorithm (VG Studio MAX, x,y,z = 3) Further, a region-growing tool was used for semiautomatic, density-based segmentation of vascular structures. Automatically segmented structures (region-growing tool) were refined manually to generate a high-resolution region of interest (ROI). Quantitative evaluation of the segmented ROI regarding vessel diameter (wall thickness analysis tool) was performed using following settings with a sphere-based method (min. 8.00 voxel, range 0.00 mm to 25 mm). Data were analyzed using GraphPad Prism (v9.4.1, GraphPad Software, San Diego, CA, USA). Results are presented as histograms, means and standard deviation (SD). Identification of the appropriate matching of the imaging details of the respective modalities (clinical CT, DVT, µCT, PBI) was done by two board-certified radiologists.

For arborization and generation analysis digitally segmented µCT and PBI datasets were reduced to 2D graphs as described previously^25^. Further analysis was performed using a customized Python v3.7 program by attributing a unique identifier number to every edge in the graph as described before (Supplementary Figure S4)^26^. Branching analyses based on the algorithms of generations, order and Strahler order were performed as described previously for lung vasculature^26–29^. In a next step, a Gaussian mixture model (GMM) was applied for clustering^30^. The number of clusters was set using the Bayesian information criterion (BIC)^60^. The Davies-Bouldin index and Dunn index were applied to compare performance of the different clustering algorithms^61,62^. Results were mapped back to the respective 3D datasets for visualization.

### Supplementary Information

Supplementary Information.

Supplementary Table S2.

Supplementary Table S3.

Supplementary Table S1.

Supplementary Video S1.

## Data Availability

The data sets generated in this study are available open access via figshare as 3D surface meshes of computed tomography (CT), digital volume tomography (DVT), micro-computed tomography (µCT) and Synchrotron-based propagation-based imaging (PBI) (https://figshare.com/articles/dataset/FileS1_surface_mesh_CT_stl/23910108; https://figshare.com/articles/dataset/FileS2_surface_mesh_DVT_stl/23910114; https://figshare.com/articles/dataset/FileS3_surface_mesh_microCT_stl/23910111; https://figshare.com/articles/dataset/FileS4_surface_mesh_Synchrotron_stl/23910117; https://figshare.com/articles/dataset/FileS5_surface_mesh_islet_Synchrotron_stl/23910105). Further information is available in the supplement to this manuscript.
