# Supplementary material for: Multiscale and multimodal imaging for three-dimensional vascular and histomorphological organ structure analysis of the pancreas
Source: Sci Rep. 2024 May 2;14:10136. doi: 10.1038/s41598-024-60254-9 (PMC11065985; doi:10.1038/s41598-024-60254-9)
Supplement: Supplementary file 1 — Supplementary Information. [file 41598_2024_60254_MOESM1_ESM.pdf]

Supporting Information

Multiscale and multimodal imaging for three-dimensional vascular and histomorphological organ structure analysis of the pancreas

Gabriel Alexander Salg, Verena Steinle, Jonas Labode, Willi Wagner, Alexander Studier-Fischer, Johanna Reiser, Elyes Farjallah, Michelle Guettlein, Jonas Albers, Tim Hilgenfeld, Nathalia A. Giese, Wolfram Stiller, Felix Nickel, Martin Loos, Christoph W. Michalski, Hans-Ulrich Kauczor, Thilo Hackert, Christian Dullin, Philipp Mayer, Hannes Goetz Kenngott*

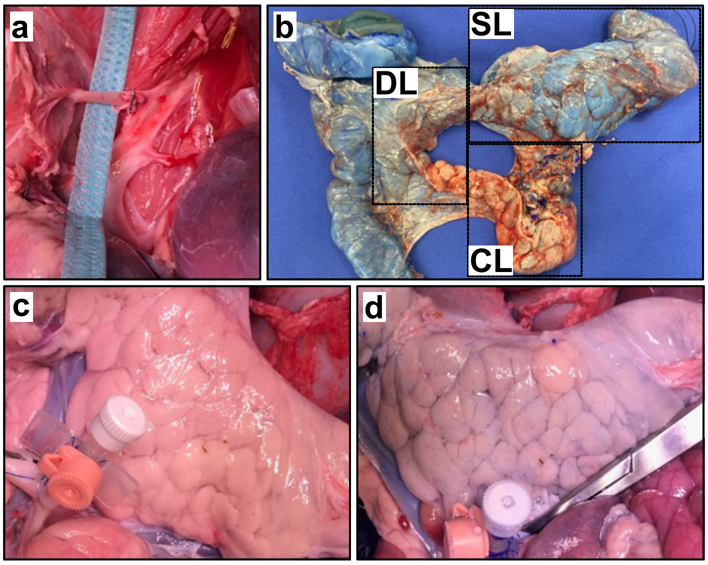

Figure S1: Macroscopic images of intraoperative situs and resected porcine pancreas. (a) Preparation of celiac trunc (looped) and superior mesenteric artery (caudally; not shown) for application of flush solution and radiopaque casting agent. (b) Resected complete porcine pancreas consisting of duodenal lobe (DL), splenic lobe (SL) and connecting lobe (CL) (including duodenum and distal stomach) after casting procedure. (c) In situ image of pancreatic tissue (SL) previous to casting procedure. (d) In situ image of pancreatic tissue immediately after start of casting agent injection via celiac trunc and superior mesenteric artery. Slight blueish color shows intravascular distribution of casting agent.

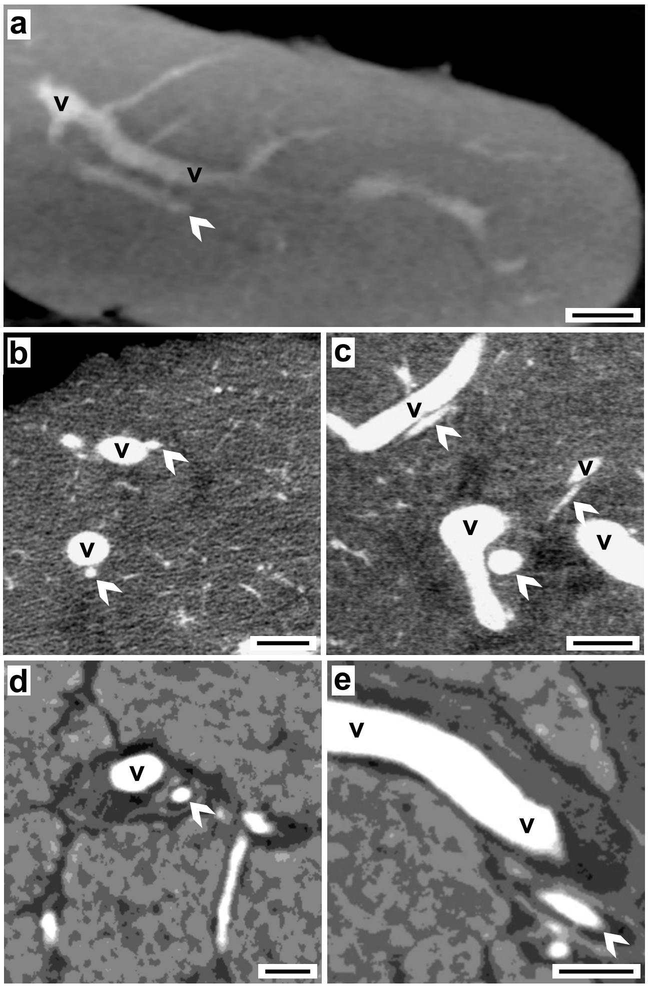

Figure S2: Imaging depicts inter-lobular veins (v) and concomitant inter-lobular arteries (arrow heads) across length scales in (a) DVT imaging and (b,c) μCT imaging. (d,e) PBI enables detection of inter-lobular venules (v) and concomitant inter-lobular arterioles (arrow heads). Scale bar 5 mm (a), 1.5 mm (b,c), 90 μm (d,e).

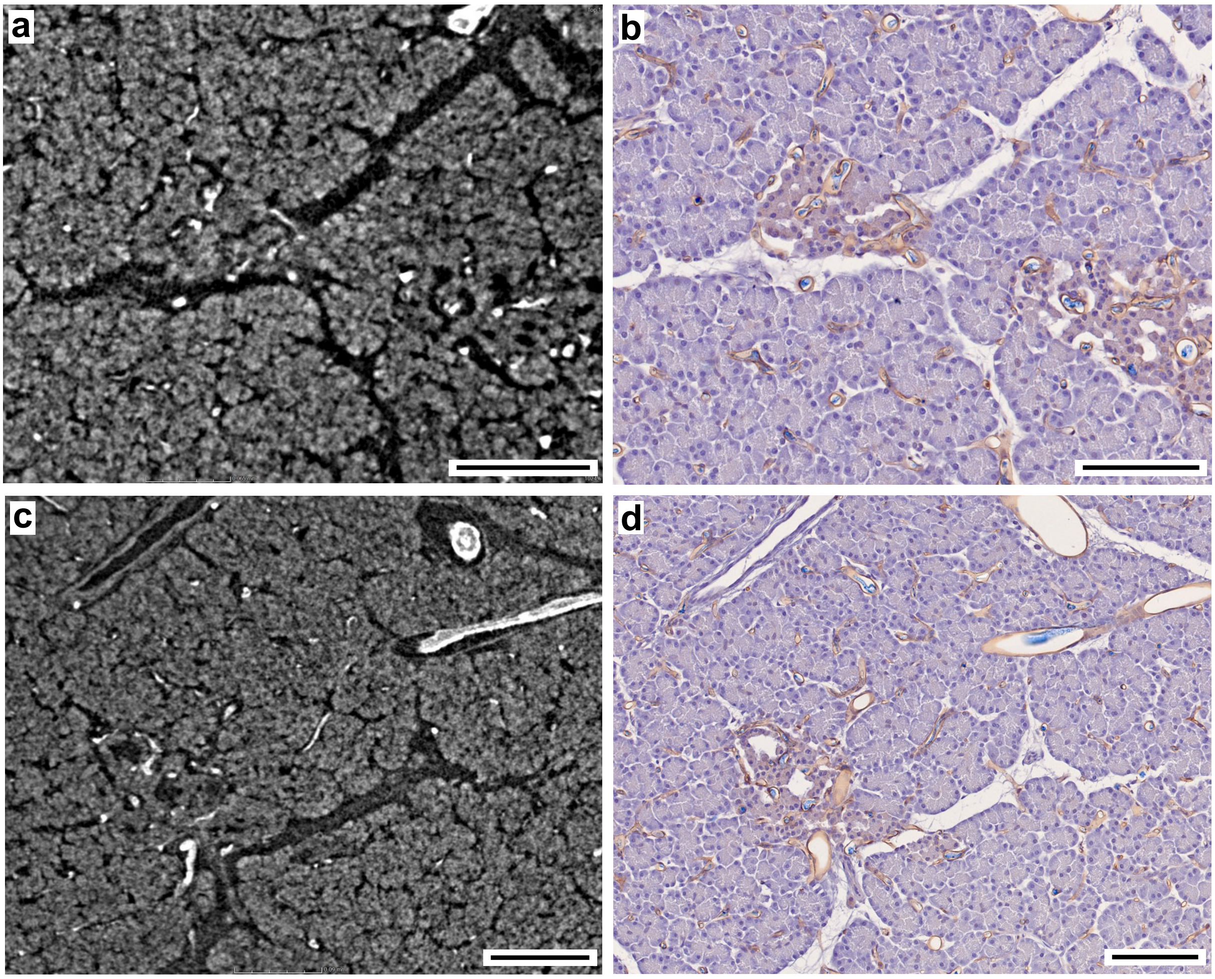

Figure S3: Synchrotron-based propagation-based imaging (PBI) correlated with tissue sections stained by immunohistochemistry. (a,c). Synchrotron-based propagation-based imaging (PBI) of tissue section with intravascular radiopaque casting, axial plane (b,d) Tissue section with anti-CD31 staining to label endothelium (brown), intravascular casting agent remnant (blue) and hematoxylin counterstaining. Scale bar 100 µm (a-d).

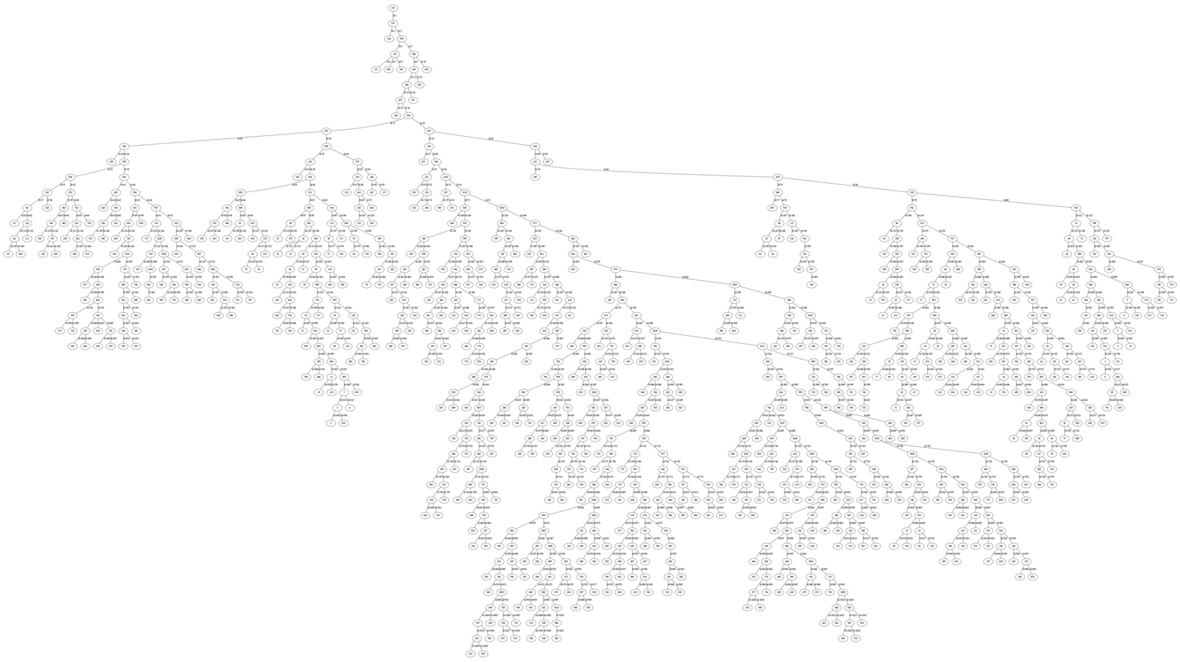

Figure S4: Arborization analysis of a representative, continuous single vessel that was digitally segmented from PBI data. The graph depicts 1024 distinct segments of the vessel. Every branching point, as well as every connecting edge are assigned identification numbers.

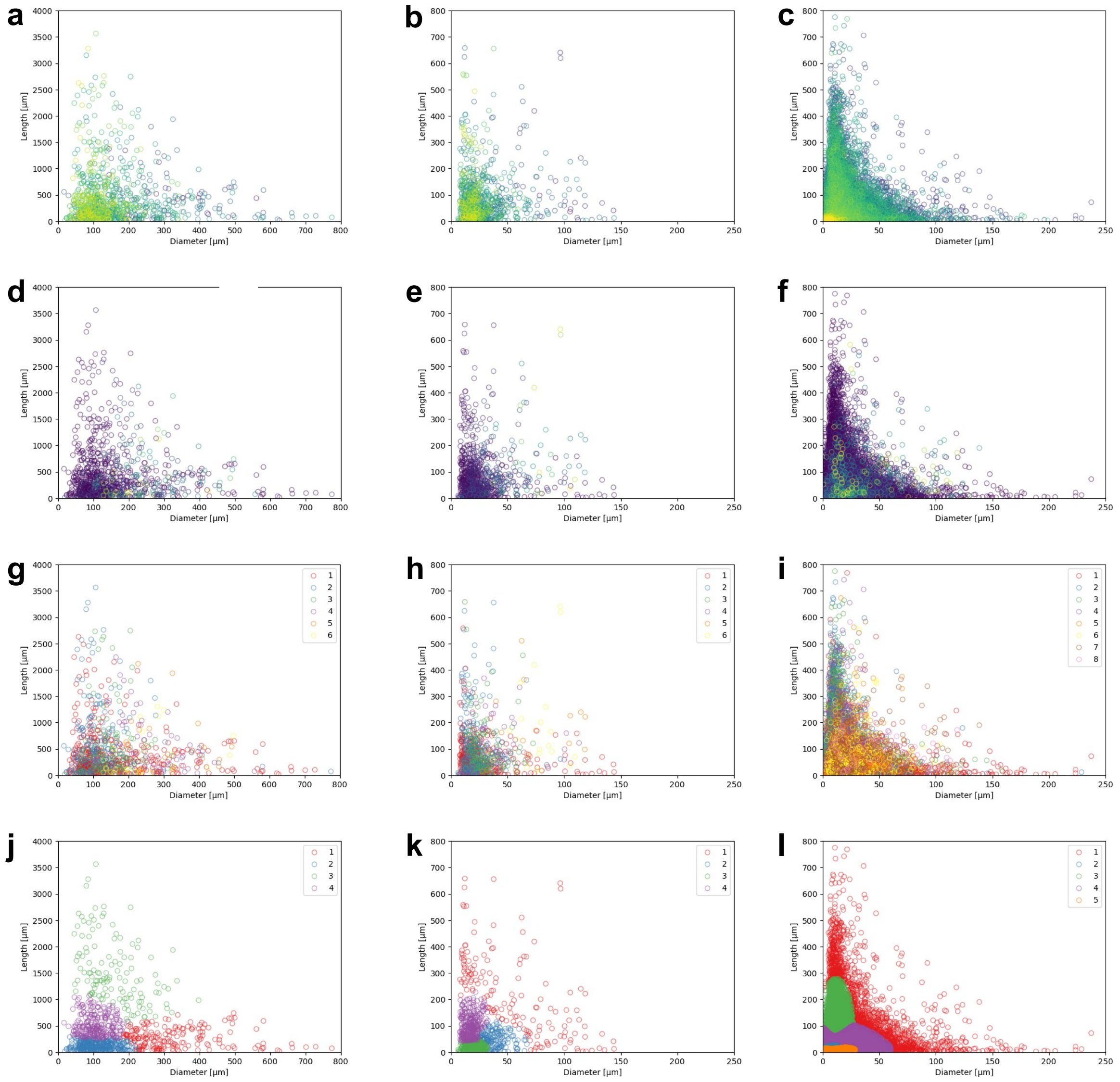

Figure S5: Application of algorithms to categorize vessel segments as (a-c) generations, (d-f) orders, (g-i) Strahler orders, (j-l) as clusters based on a GMM. In the left column (a,d,g,j) a single, continuous vessel obtained from µCT data was analyzed. In the middle column (b,e,h,k) a single, continuous vessel obtained from PBI was analyzed. In the right column (c,f,i,l), data obtained from a digitally segmented vascular network from PBI data are plotted. (a) 81 generations, (b) 42 generations, (c) 293 generations, (d) 81 orders, (e) 42 orders, (f) 293 orders, (g) 6 Strahler orders, (h) 6 Strahler orders, (i) 8 Strahler orders, (j) 4 GMM clusters, (k) 4 GMM clusters, (l) 5 GMM clusters.

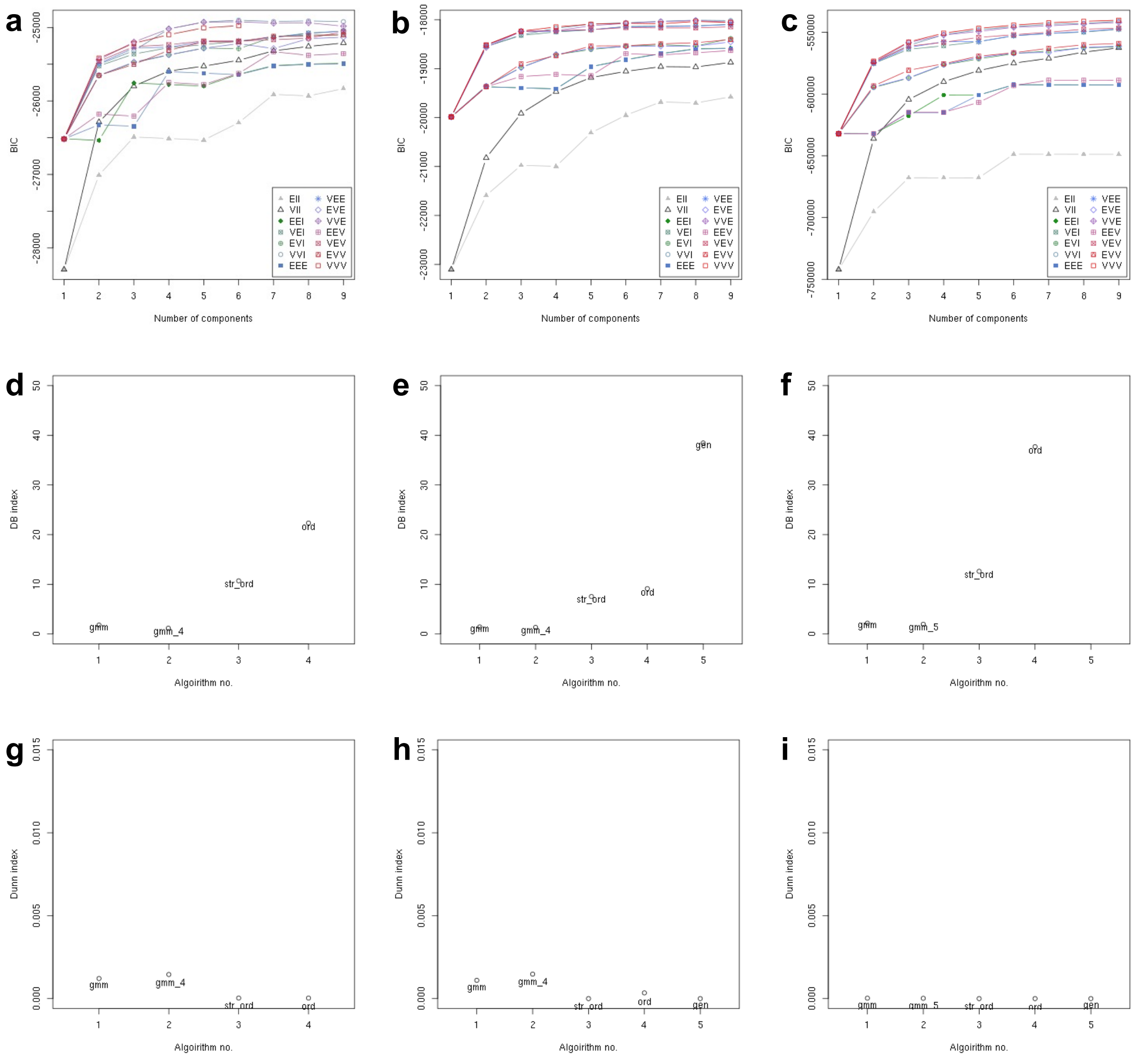

Figure S6: (a-c) Bayesian information criterion (BIC) for cluster counts 1-9 and 14 geometric constellations. (d-f) Davies Bouldin index and (g-i) Dunn index for GMM clustering and generation, order and Strahler order algorithms. In the left column (a,d,g) data from a single, continuous vessel obtained from µCT were analyzed. In the middle column (b,e,h) data from a single, continuous vessel obtained from PBI were analyzed. In the right column (c,f,i), data obtained from a digitally segmented vascular network from PBI data are plotted. → not finalized

Modality	Object	Generation	Order	Strahler order
**µCT**	**Single vessel**	81	81	6
**PBI**	**Single vessel**	42	42	6
**PBI**	**Tissue section**	293	293	8

Table S1: Overview of results from vessel segment analysis using generation, order and Strahler order algorithms.

Modality	Object	Algorithm	Davies-Bouldin index	Dunn index
**µCT**	**Single vessel**	**Generation**	41.7	<0.1
		**Order**	27.0	7.4
		**Strahler order**	20.6	7.5
		**Clustering (GMM)**	1.0	7.5
**PBI**	**Single vessel**	**Generation**	37.1	<0.1
		**Order**	12.3	<0.1
		**Strahler order**	11.3	<0.1
		**Clustering (GMM)**	1.2	<0.1
**PBI**	**Tissue section**	**Generation**	162.0	<0.1
		**Order**	43.6	<0.1
		**Strahler order**	15.0	<0.1
		**Clustering (GMM)**	1.8	<0.1

Table S2: Calculation of Davies Bouldin index and Dunn index to evaluate clustering and conventional grouping performance.

s. separate file for Table S3

Table S3: Detailed results from vessel segment analysis using Strahler order and GMM clustering (per group/per cluster).

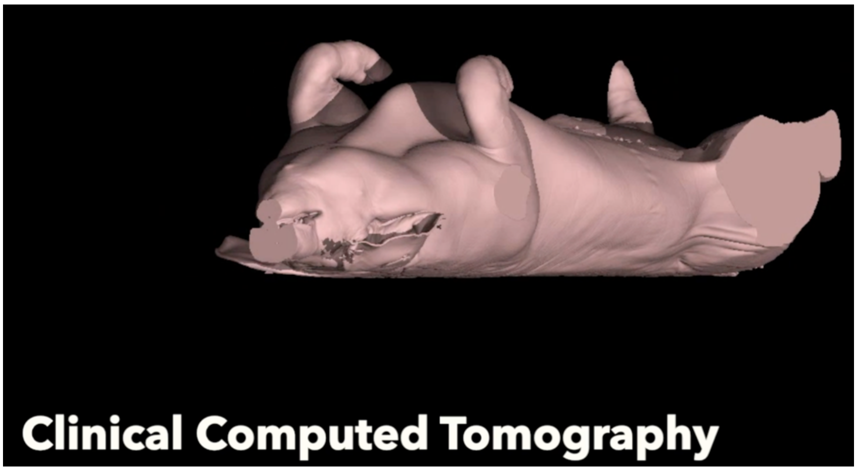

Video S1: Imaging cascade and histological correlation: Vascular network and islets of Langerhans in the porcine pancreas. Video file can be found in the attachment to this manuscript.

File S1: 3D-surface mesh of peri- and intrapancreatic vascular network imaged by computed tomography. Open access data publication: doi.org/10.6084/m9.figshare.23910108

File S2: 3D-surface mesh of peri- and intrapancreatic vascular network imaged by digital volume tomography. Open access data publication: doi.org/10.6084/m9.figshare.23910114

File S3: 3D-surface mesh of vascular network imaged by micro-computed tomography. Open access data publication: doi.org/10.6084/m9.figshare.23910111

File S4: 3D-surface mesh of of vascular network imaged by Synchrotron-based propagation-based imaging (PBI). Open access data publication: doi.org/10.6084/m9.figshare.23910117

File S5: 3D-surface mesh of peri- and intra-islet vascular network imaged by Synchrotron-based propagation-based imaging (PBI). Open access data publication: doi.org/10.6084/m9.figshare.23910105
